# Even more suicide attempts in clinical trials with paroxetine randomised against placebo

**DOI:** 10.1186/1471-244X-6-55

**Published:** 2006-11-28

**Authors:** Ivar Aursnes, Ingunn Fride Tvete, Jorund Gaasemyr, Bent Natvig

**Affiliations:** 1 Department of Pharmacotherapeutics, University of Oslo, Oslo, Norway; 2 Department of Mathematics, University of Oslo, Oslo, Norway

## Abstract

**Background:**

Following our previous publication we have received critical comments to our conclusions as well as new data that are strengthening our findings.

**Results:**

With the new data, 11 suicide attempts among patients on paroxetine against 1 among patients on placebo, we found with a Bayesian technique that the posterior probability that medication with paroxetine is associated with an increased intensity per year of a suicide attempt is from 0.98 to 0.99, depending on the prior.

We found that the comment to our article by GSK representatives contained errors, misunderstanding and unwillingness to accept Bayesian principles in the analysis of clinical trials.

**Conclusion:**

We were in our previous publication, with preliminary data and a Bayesian approach, able to raise a concern that suicide attempts might be connected with the use of paroxetine. This suspicion has now been confirmed.

## Text

Last year we wrote a paper "Suicide attempts in clinical trials with paroxetine randomised against placebo" [1] that hit the front pages of newspapers worldwide [2,3]. Our publication demonstrated an increased intensity of suicide attempts per year when using paroxetine compared to placebo, and caused GlaxoSmithKline (GSK) to come up with a comment [4]. Since then GSK has provided additional data to the American Food and Drug Administration (FDA), as the agency required new documentation on paroxetine. This also resulted in a Briefing Document from GSK [5] in which they admit that there is an increased risk for suicide attempts associated with paroxetine.

**Figure 1:**
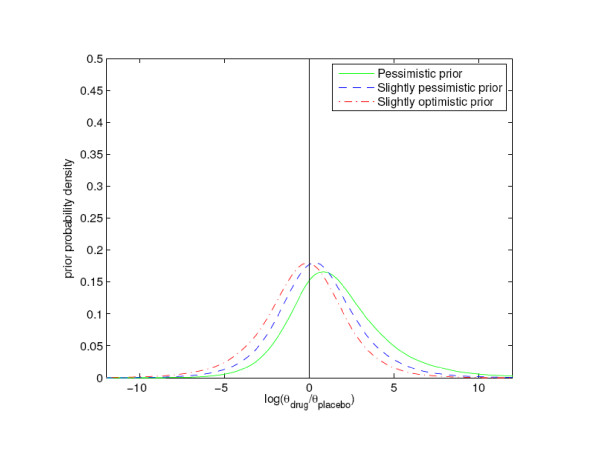
**Prior intensity of suicide attempt**. Distributions of three different prior (see text) logarithmic intensity ratios log(θ_drug_/θ_placebo_) (logarithmic intensity of a suicide attempt on drug minus logarithmic intensity of a suicide attempt on placebo).

**Figure 2:**
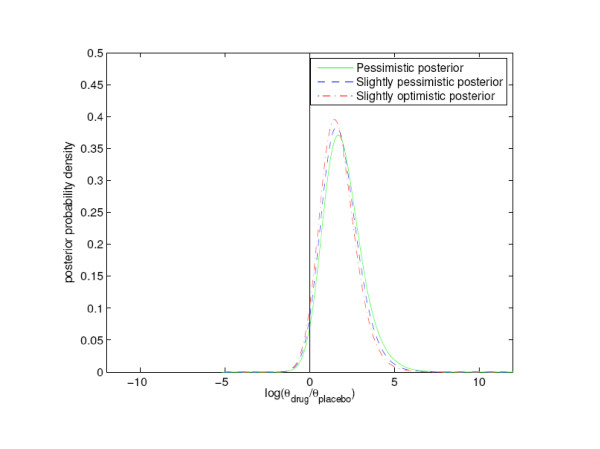
**Posterior intensity of suicide attempt**. Distributions of posterior logarithmic intensity ratios log(θ_drug_/θ_placebo_) with three different priors. .

We analyzed the data GSK presented in their latest report by the same Bayesian approach used by us in our article. We included only the double blind, parallel design studies with patients randomized to either paroxetine or placebo, as recognized by GSK in the Briefing Document. These 19 studies contained 3455 and 1978 patients to the treatment and placebo groups, respectively. They resulted in 11 and 1 suicide attempts, respectively, as compared to 7 and 1 in our study. The studies lasted 6 – 12 weeks, and we obtained 601 and 333 patient years in the treatment and placebo groups, respectively. Since we did not have access to the points of time of censoring, we were not able to correct for this when computing the patient years. We took the same model approach as in our article, and refer to it for details regarding the model. We let θp and θd be the mean number, or intensity, per year of a suicide attempt for a random patient in the 19 studies in the placebo and treatment group, respectively. We performed simulations by making 80000 random draws of θp and θd, from their independent posterior distributions. We computed the logarithms of the ratios θd/θp, and constructed posterior probability density plots by applying a standard density estimation technique to these 80000 numbers. (The logarithm was introduced to avoid an unwelcome feature of the density estimation method). Note that the logarithm of the ratio θd/θp is greater than zero whenever θd is greater than θp. Hence, we calculated the probabilities that medication with paroxetine is associated with an increased intensity of a suicide attempt per year as the proportions of logarithmic ratios greater than zero in the samples. We applied three different prior assumptions based on earlier work by others, as described in the article. We chose to express the pessimistic view as equivalent to observing 0.86 events with paroxetine during 50 patient years and 0.43 with placebo during 50 patient years, adding up to 1.29 attempts per 100 patient years, which is similar to the observed average value for paroxetine and placebo taken together. We based the calculations on a total of only 100 patient years in the prior, compared to 934 patient years in the real data, in order to increase the importance of the real data over the prior information. For the slightly optimistic and slightly pessimistic priors we assigned the numbers of suicidal patients on paroxetine and placebo per 50 patient years to be respectively 0.58 and 0.71 and vice versa. We found that the posterior probability that medication with paroxetine is associated with an increased intensity per year of a suicide attempt is 0.99 with the pessimistic prior, 0.98 with the slightly optimistic prior and 0.99 with the slightly pessimistic prior. Hence, we can be at least 98 % sure that paroxetine increases suicide attempts. This is stronger evidence than the p value equal to 0.058 given in the Briefing Document [5]. The corresponding prior probabilities were respectively 0.70, 0.44 and 0.56. See figures 1 and 2 for illustrations.

In addition to calling into question the statistical method applied, the authors of the GSK comment denied our findings by disputing the selection of data. They state that "analysis was based solely on early data submitted to regulatory authorities in 1989. The studies analyzed included only 916 paroxetine-treated patients. Today we have data from more than 13,000 adult patients treated with paroxetine in well-controlled clinical trials." Interestingly, GSK has now in the Briefing Document reduced the corresponding number to 3455 and also admitted that they previously had several patients with a history of suicide attempts from an earlier trial placed in the treatment and placebo groups. With such a case history these patients were more likely to attempt suicide again. They should obviously not have been included since they blurred the results. This can be described as 'data-drowning', and is at least poor methodology.

One item in the comment by GSK concerns two cases that they believe we had misplaced. One patient (no 1.12.037) was claimed to show only suicidal ideation but has by GSK in the Briefing Document been re-classified to represent a suicide attempt. The other patient (no 04.02.056) was from a cross over continuation study. We do not agree that this patient should have been excluded.

GlaxoSmithKline state in their comment to our article that the risk may be closely related to the point of time observed in a study, and further that they would expect to have 6 attempts in the treatment group out of a total events of 8. The number is more precisely 5.79. Hence, our number 7 represents a 20.9 % increase compared to what is expected. They apply a Fishers Exact Test, which in our opinion is a very coarse method. Both in their earlier report in which they applied the Mantel-Haenszel test, and in their new analysis in the Briefing Document [5] where they applied a so-called exact method, they do not take the exposure time of the patients properly into account, as we have done.

In the comment to our article it was claimed that we should have compared a hypothesis of an increased number of suicide attempts to one in which the rate is identical for the treatment group and placebo group. This hypothesis is meaningless. No one believes that the rates for these two groups are exactly equal. Secondly, we do not in any way attempt to do any form of hypothesis testing. We are merely computing the probability that medication with paroxetine is associated with an increased intensity per year of suicide attempts. GSK questions our statement that '...the data strongly suggest that the use of SSRIs is connected with an increased intensity of suicide attempts per year'. We feel that this is all about understanding and accepting the Bayesian approach as a way of analysing data. Recently FDA issued a drafted guidance for using Bayesian statistics in clinical trials [6]. They write 'Bayesian statistics is a statistical theory and approach to data analysis that provides a coherent method for learning from evidence as it accumulates.' Hopefully FDA's guidelines will contribute to an increasing understanding of the Bayesian approach.

We conclude that irrespective of the various prior assumptions, we now see clearer that the data strongly suggest that the use of antidepressant drugs is connected with an increased intensity of suicide attempts per year, not only among young people, but also among adults. Moreover, information from studies on antidepressants and other drugs must be made open for researchers and presented in the most informative way possible. We believe that this would create an open minded, trusting dialogue between consumers, manufacturers and scientists working towards the same goal: finding the best and safest treatment for the patients.

## Competing interests

The author(s) declare that they have no competing interests.

## Authors' contributions

IA collected the data, presented the problem and drafted the manuscript along with BN, who suggested the statistical solution based on earlier work by the present authors [7]; IFT did the computations and took part in the planning along with JG. All authors read and approved the final manuscript.

## Pre-publication history

The pre-publication history for this paper can be accessed here:


